# Updated fraction of cancer attributable to lifestyle and environmental factors in Denmark in 2018

**DOI:** 10.1038/s41598-021-04564-2

**Published:** 2022-01-11

**Authors:** Anne Julie Tybjerg, Søren Friis, Katrina Brown, Mef Christina Nilbert, Lina Morch, Brian Køster

**Affiliations:** 1 Research Center, Danish Cancer Society, Strandboulevarden 49, 2100 Copenhagen Ø, Denmark; 2 Cancer Intelligence Team, Cancer Research UK, London, UK; 3 Department of Oncology, Institute of Clinical Sciences, Lund University, Lund, Sweden; 4 Cancer Prevention and Information, Danish Cancer Society, Copenhagen, Denmark

**Keywords:** Cancer epidemiology, Cancer prevention, Cancer, Risk factors

## Abstract

Environmental exposures and avoidable risk factors account for a large proportion of cancer burden. Exposures and lifestyle vary over time and between populations, which calls for updated and population-specific quantification of how various avoidable risk factors influence cancer risk to plan and design rational and targeted prevention initiatives. The study considered 12 risk-factor groups categorized as class I carcinogens by IARC/WCRF. Exposure data was derived from national studies and surveys and were linked to cancer incidence in 2018 based on the nationwide Danish Cancer Registry. In 2018, 23,078 men and 21,196 women were diagnosed with cancer excluding non-melanoma skin cancer, in Denmark. Of these, 14,235 (32.2%) were estimated to be attributable to avoidable class I carcinogens. Tobacco smoking accounted for 14.6% of total cancers, followed by UV-radiation that accounted for 5.8%. Based on exposure data from 2008, one-third of the cancers in Denmark in 2018 are estimated to be caused by class I carcinogens with tobacco use being the main contributor followed by UV-radiation. Our results should be integrated with public health policies to effectively increase awareness and promote strategies to decrease risk factor exposures at population level.

## Introduction

The global number of patients with cancer is expected to double over the next 50 years. To counteract this trend evidence-based and comprehensive cancer prevention strategies are needed to reduce the prevalence of risk factors and thereby the number of future cancer cases. National cancer control programs define evidence-based services and actions within primary prevention and health promotion with the aim of mitigating risk factors and cancer burden through e.g. public policies, health promoting environments, personal and community actions and optimized health services. Prompt implementation, focus on equity and prioritization of efforts have been linked to public health advantages and economic benefits^1,2^.

Incidence patterns vary between cancer types with increasing incidence in Denmark for e.g. brain tumors, kidney cancer, malignant melanoma of the skin and female thyroid cancer, whereas decreasing incidences are documented for ovarian cancer, lung cancer in men and pancreatic cancer in women^3,4^. Future cancer trends depend on national health policy and trends in risk factors. Depending on tobacco policies introduced in the Nordic countries in 2016, the projection of tobacco related cancer cases in 2045 can be estimated to be reduced by 5% for continued tobacco reduction trend, 8% for taxation and restrictions and 19% for complete elimination of tobacco^5–8^.

Cancer predisposing and initiation of DNA damage primarily stem from replicative, hereditary, and environmental factors. Estimates on 17 cancer types in 69 countries suggests that 42% of cancer cases would have been avoidable if known environmental risk factors had been eliminated^9^. Several modifiable risk factors for cancer have been identified and include tobacco smoking, obesity and overweight, occupation, infections, alcohol consumption, ionizing radiation, processed meat, air pollution, physical activity, UV-radiation, post-menopausal hormones and oral contraceptives^10^. Data on how various risk factors influence cancer risk and the prevalence of these risk factors in the population allow for estimation of population attributable fractions (PAF) of cancer^11^. For UK, Parkin et al.^12^ and Brown et al.^10,12^ estimated the burden of theoretically avoidable cancer in 2010 and 2015, respectively. While Denmark and UK are comparable neighbor countries, differences in risk factor prevalences exist, e.g., Denmark has higher smoking prevalence, while the UK has a higher prevalence of obesity, and PAF estimates have revealed substantial differences between various countries for specific risk factors, cancer types and combinations hereof^13–15^.

New high-quality evidence on relative risks, demographic changes among cancer patients, and changes in official classifications of risk factor evidence strength, as governed by the International Agency for Research on Cancer [IARC] and World Cancer Research Fund [WCRF], may alter PAFs and thus prioritizations. For classification of class I/convincing cancer risk factors we applied the term ‘class I carcinogen’ in the present study. We provided near time estimates of PAFs by cancer type and risk factors based on a population approach with complete data on exposure to class I carcinogens and cancer incidence in the Danish population.

## Materials and methods

We designed a population-based prospective study that linked risk factor exposures in the 2008 to cancer incidence a decade later, i.e. in 2018. The PAF estimates were based on the methodology developed by Parkin et al.^10,12^.

$$\frac{{\left( {p_{1} \times {\text{ERR}}_{1} } \right) + \left( {p_{2} \times {\text{ERR}}_{2} } \right) + \left( {p_{3} \times {\text{ERR}}_{3} } \right) \cdots + \left( {p_{n} \times {\text{ERR}}_{n} } \right)}}{{1 + \left[ {\left( {p_{1} \times {\text{ERR}}_{1} } \right) + \left( {p_{2} \times {\text{ERR}}_{2} } \right) + \left( {p_{3} \times {\text{ERR}}_{3} } \right) \cdots + \left( {p_{n} \times {\text{ERR}}_{n} } \right)} \right]}}$$

PAF defines the contribution from each risk factor to disease development on the population level. The PAF estimates enable quantification of how a change in prior exposure to cancer risk factors affects the current incidence of cancer in the population under the assumption that all other factors remained constant. To assess this, we used a standard formula where *p* is the proportion of the population in each exposure level (1, 2, … *n*) and excess relative risk (ERR) (the relative risk (RR) − 1) at each exposure level (1, 2, … *n*).

PAFs calculated based on reduced incidence or absence of a given risk factor were based on the RRs for the presence of or higher level of the specific factor. The ERRs were calculated as the natural logarithm of the reciprocal of the RR (ln(1/RR) as described by Brown et al.^10^. For risk factors with multiple units, the ERR was calculated for each unit by division with the number of units.

For the risk factors Epstein–Barr virus (EBV), human papillomavirus (HPV), occupation, and UV-radiation, we applied PAF estimates from published studies, as more accurate data were available in these publications than could be obtained based on Danish registry data^10,16,17^, Supplementary Material C and E.

### Risk factors and cancer types considered

Risk factors that were defined as class 1 carcinogens were eligible for the study. As an entry point, we used the same risk factors as reported in the UK population study by Brown et al.^10^, with modification related to breastfeeding and intake of dietary fiber since these factors have, based on new evidence, been downgraded from ‘convincing’ to ‘probable’ and are thus not class 1A carcinogens^18^. In total, 12 class I carcinogens were considered (Supplementary Material C).

Consideration of specific cancer types for the various risk factors were based on causal associations classified as “sufficient” according to IARC^19^ and/or “convincing” according to WCRF^18^ as of January 2021. *I.e. an association could be categorized as probable by WCRF but still be included if classified class I by IARC and *vice versa* have a lower categorization by IARC and still be included if WCRF categorized it as convincing.* Classification details are summarized in Supplementary Material A. We solely considered the negative impact of each risk factor, which implies that a potential positive impact of e.g. oral contraceptives was not considered (Supplementary Material A and D).

#### PAF

The RRs included in our analysis are primarily based on the risk estimates described by Brown et al.^10,12^. Literature searches were similarly updated to establish whether updated evidence has emerged since April 2017^10^. Risk estimates based on more recent studies and/or based on population-specific estimates from Denmark were prioritized for the calculations. This applied for colon cancer and rectal cancer in relation to processed meat (Supplementary Material D). Multiple risk factors and individual factors may interact to define risk of cancer and PAFs for individual risk factors may overlap and exceed 100%, which motivated adjustment in the PAF estimates, as described by Brown et al.^10^ In short, to avoid overestimating the combined number of attributable cases, the combined PAF takes cases explained by another risk factor into account (Supplementary Material E and F).

### Exposure prevalence and cancer incidence

Data on exposure prevalence were collected from Danish surveys and registry studies using exposure data as close to 2008 as possible (Supplementary Material C). The cancer incidence in Denmark in 2018 was based on the annual incidence reported from the Danish Cancer Registry^20^ (Supplementary Material B). All cancer types, except non-melanoma skin cancer, were considered, which translated to International Classification of Diseases version 10 [ICD-10] codes C00-C97, except C44. The Cancer Registry defines incidences by a 3-digit ICD-10 code, while other cancer types were categorized according to Danish standards. In cases where the risk estimates cover cancer types defined differently from main categories, we used individual-level data from the Danish Cancer Registry to calculate the incidence of the specific cancer type or subtype in 2018. Further, risk estimates based on certain subgroups, e.g. related to histologic subtype or age group, were considered and accounted for in the calculations. Diagnoses with an annual population incidence below 10 were excluded.

The study was performed in accordance with the Declaration of Helsinki. According to Danish law, ethical approval is not required for epidemiologic register studies.

## Results

**Figure 1:**
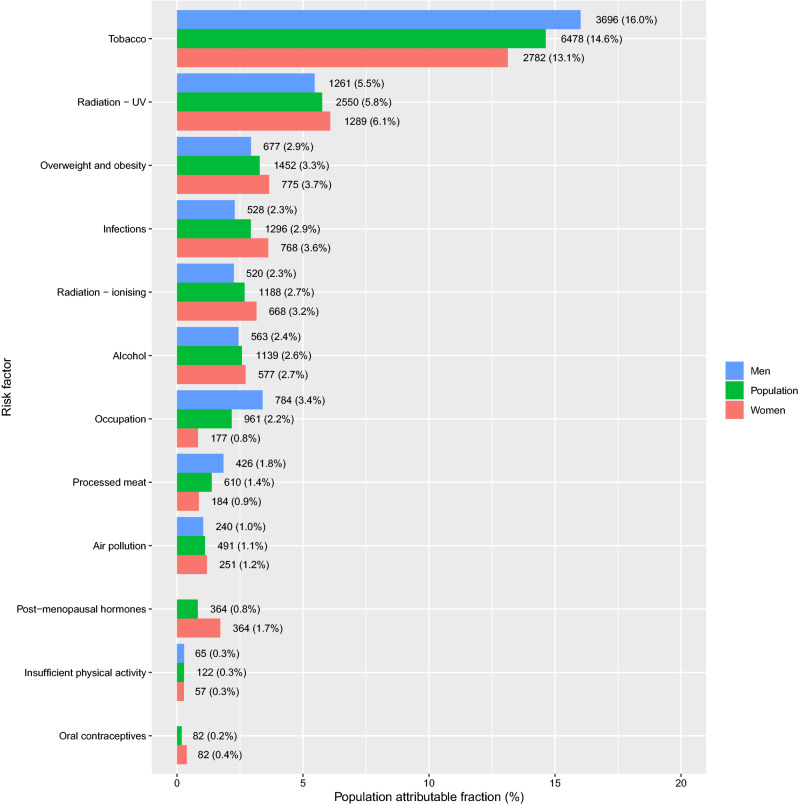
Population attributable fraction of cancer in Denmark in 2018 distributed on known class I carcinogen risk factors. The total number of cancers in Denmark in 2018 was 14,235 and the fraction of attributable cancers to known risk factors was 32.2%.

**Figure 2:**
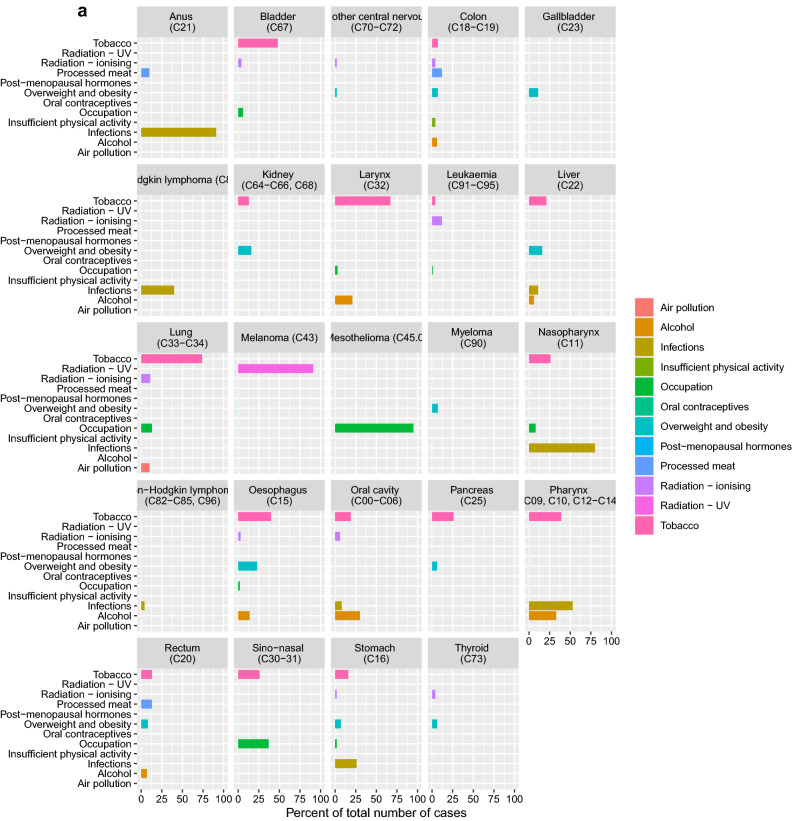

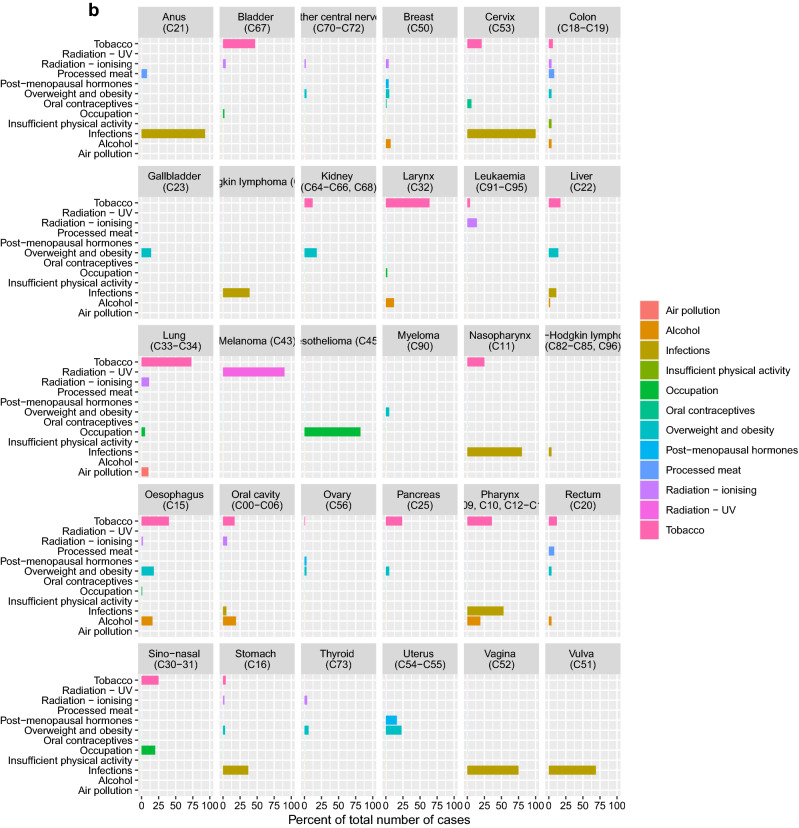

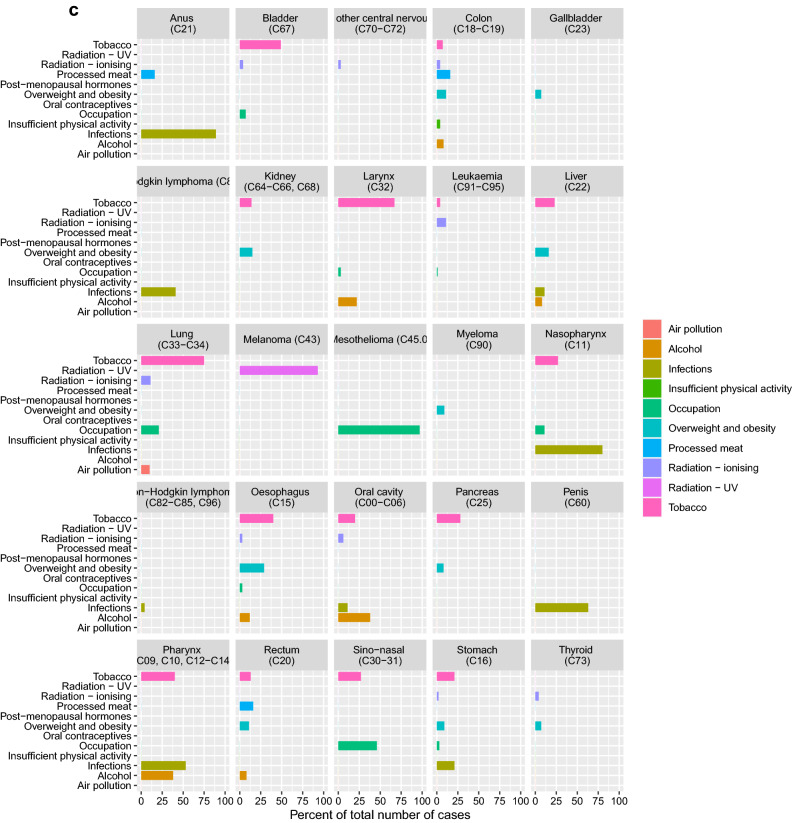
(**a**) Population attributable fraction of cancer among women and men in Denmark in 2018 distributed on cancer type and known class I carcinogen risk factors. (**b**) Population attributable fraction of cancer among women in Denmark in 2018 distributed on cancer type and known class I carcinogen risk factors. (**c**) Population attributable fraction of cancer among men in Denmark in 2018 distributed on cancer type and known class I carcinogen risk factors.

In 2018, 23,078 men and 21,196 women were diagnosed with cancer (excluding non-melanoma skin cancer) in Denmark. Based on the 12 groups of class I carcinogenic factors, 32.2% of all cancer cases were estimated to be attributable to these exposures and life-style factors. The proportion was similar in males (31.2%) and in females (33.2%). PAFs and attributable cases, by risk factor and sex are presented in Fig. 1. Detailed results by risk factor–cancer type combination, cancer type, and sex are presented in Fig. 2a–c and in Supplementary Material E.

Tobacco smoking contributed the largest proportion of attributable class I carcinogen cases, accounting for 14.6% of all cancer cases. The proportion was higher in males (16.0%) than in females (13.1%), (Supplementary Material C). Of the 17 cancer types with an established link to tobacco smoking, the highest PAFs applied to lung cancer (73.9%) and laryngeal cancer (66.8%). The highest attributable numbers applied to cancers of the lung (n = 3612) and urinary bladder (n = 955).

UV radiation contributed the second-largest overall proportion of attributable cancer cases in 2018 with a PAF of 5.8% in the population (5.5% in males and 6.1% in females). The PAF for melanoma skin cancer was 93.2% for men and 89.5% for women.

Overweight and obesity combined was the third-largest preventable cause of cancer accounting for 3.3% of all cancer cases. The proportion was slightly higher in females (3.7%) than in males (2.9%), despite a higher prevalence of overweight and obesity among men (40.5%) compared to women (26.3%). The cancer types with the highest PAFs for overweight and obesity were oesophageal cancer (23.3%), endometrial cancer (22.6%), liver cancer (15.7%), and kidney cancer (16.1%). The highest attributable number of cases applied to colon cancer (246) and breast cancer (n = 235).

Infectious agents, i.e. HPV, Helicobacter Pylori, Hepatitis B and C, Human Immunodeficiency Virus (HIV) and EBV, combined contributed 2.9% of all cancers. The proportion was considerably higher in females (3.6%) than males (2.3%). The total number of cases attributable to infections was 1,296. For HPV, the highest PAFs were seen for anal cancer (91.5%) and cervical cancer (99.7%). The highest attributable numbers for infectious agents combined applied to cervical cancer (n = 332) and pharyngeal cancer (n = 255).

Ionising radiation accounted for 2.7% of all cases (n = 1188), of which 420 were attributable to radon exposure and 768 were related to other sources of radiation: medical exposure and background radiation (cosmic, food intake, earth crust). The most frequent cancer type attributable was lung cancer (n = 608).

Alcohol consumption accounted for 2.6% of all cancers (2.4% for men and 2.7% for women) and was responsible for n = 1,139 attributable cancer cases. The highest PAF was observed for oral cavity cancer (30.3%) and pharyngeal cancer (33.0%) while the highest number of attributable cases were seen for breast cancer (n = 370) and colon cancer (n = 206).

Occupational exposures to class I carcinogens were estimated to account for 2.2% of all cancer cases in 2018 with a significantly higher proportion in men (3.4%) than in women (0.8%), which is explained by differences in PAFs as well as incidence of associated cancer types such as urinary bladder cancer and mesothelioma (pleural). Occupational factors were associated with an increased risk for 15 cancer types with lung cancer, mesothelioma and urinary bladder cancer accounting for > 90%. The highest number of cancers attributable to occupational exposures applied to lung cancer (n = 504 in men and n = 132 in women).

Processed meat accounted for 1.4% of all cancers and was related to colon cancer and rectal cancer. The proportion of attributable cases was twice as high in men (1.8%) as in women (0.9%), reflecting a lower intake of processed meat in women and a lower relative risk of colorectal cancer. Processed meat was estimated to cause n = 610 cancer cases.

Air pollution contributed to 1.1% of all cancers (1.0% for men and 1.2% for women). The slight differences between the sexes that may be explained by a higher lung cancer incidence in women.

Postmenopausal hormones accounted for 1.7% of female cancers with the highest PAF, 16.4%, for endometrial cancer while the highest number was attributable to breast cancers (n = 219).

Insufficient physical activity is associated with an increased risk of colon cancer and was estimated to contribute to 0.3% of all cancers in both men and women with an annual number of attributable colon cancers of n = 65 in men and n = 57 in women.

Oral contraceptives contributed 0.4% of all female cancer cases and accounted for n = 82 breast cancers and cervical cancers.

Four of the five most incident cancer types in Denmark, i.e. breast cancer, lung cancer, colon cancer and malignant melanoma, showed significant PAFs, translating to a total of 8617 avoidable cases annually (Supplementary Material E). Breast cancer was linked to overweight and obesity, alcohol, ionizing radiation, postmenopausal hormone therapy and oral contraceptives with 19.9% (n = 993) avoidable cases. Lung cancer attributed to class 1 risk factors such as tobacco, occupational exposures, ionizing radiation, and air pollution comprised 81.8% (n = 3995) avoidable cases. Colon cancer was associated with smoking, overweight, alcohol radiation, intake of processed meat, and insufficient physical activity with 32.6% (n = 1079) avoidable cases. Malignant melanoma of the skin was associated with UV radiation with 91.3% (n = 2550) avoidable cases.

## Discussion

Based on Danish data, we estimate that 31.7% of cancers diagnosed in the population in 2018 were attributable to known preventable class I risk factors as defined by the WHO and/or WCRF. In absolute numbers, this corresponds to more than 14,000 cancer cases for Denmark, which could potentially have been avoided. Tobacco smoking was by the far largest preventable cause of cancer (14.6%) followed by UV-radiation (5.8%) and overweight/obesity (3.3%). While tobacco smoking does not present the highest population-level exposure, the very high relative risks and many cancer types associated with this exposure explains the position of tobacco as the leading cause of cancer in Denmark as well as most other high human development index countries.

The predominant PAF for tobacco smoking of 14.6% was somewhat lower than previously estimated, e.g. 20% in Denmark in 1990 when 40% of the population were smokers^11^, but comparable to the 15% estimated in the UK with 25% smokers in 2005^10^. Also in countries with low smoking prevalence, tobacco remains the predominant PAF as recently demonstrated in a report from Sweden with < 10% daily smokers^21^.

The PAF for UV-radiation was among the highest worldwide and at the level of countries like Australia and Sweden^10,21–23^. Denmark has the third highest melanoma incidence worldwide, which is linked to fair skin types and sun-seeking behavior with intermittent UV-exposures, use of sunbeds and vacations to destination with UV-index levels not adapted for^3^. The increasing melanoma incidence is the main cause of the increasing PAF, however, refined diagnostic possibilities may have contributed to the observation^24^. Though the Danish Cancer Registry contains information on non-melanoma skin cancer, we did not include these diagnoses to achieve comparative study designs.

The PAFs for dietary factors were considerably lower than previously suggested^10,18,22^. However, this finding was primarily due to reclassification of fruit/vegetables, fiber, and red meat currently classified as class 2 evidence according to the IARC and WCRF.

The total PAF was largest for men who had a higher exposure to the majority of the included established preventable risk factors such as tobacco smoking, alcohol consumption, intake of processed meat, overweight and obesity, UV-radiation and occupational exposure. than women (Supplementary Material C). Gender differences in associated relative risk to the exposure, differences in cancer incidences and gender specific cancers, however, resulted in a higher PAF for UV-radiation and overweight and obesity, infections, and alcohol consumption for women. Considerable differences apply in alcohol intake between men and women in Denmark with five times as many men having a median daily intake of alcohol above 50 g compared to women (Supplementary Table C). However, since drinking increases the risk of female breast cancer, which is the most common cancer type in Denmark, this explains why women overall have a higher proportion of cancers attributable to alcohol. Obesity and overweight showed higher overall PAFs in women, which is explained by associations between overweight and obesity and several female cancer types such as ovarian cancer, breast cancer, and endometrial cancer. In addition, infections agents showed higher PAFs in women, which can be explained by the link between HPV infections and female cancers such as cervical cancer, vulvar cancer and vaginal cancer.

PAF estimates from the UK, Canada, Sweden and China report 28–45% cases linked to avoidable risk factors^10,12,25,26^. The studies are not readily comparable due to differences in methodology, study time periods, population origins, modifications in cancer classification and selection of risk factors^27^. Parkin et al.^10^ and Grundy et al.^26^ previously reported estimates of more than 40% for PAFs of the cancer burden in UK and Alberta, Canada, respectively, although the studies also considered intake of vegetables and vitamin D. The highest reported PAF of 45% derives from China with strong influence from viral infections and vegetable intake^23,28^. Brown et al.^10^ estimated an overall PAF for cancer burden of 37.7% in the UK based on 2015 data^18^. This study also considered the factors low dietary fiber intake and breastfeeding, which have now been downgraded from convincing to probable, and the former factor alone contributed to over 3% of the cancer burden. UK has a higher proportion of obese or overweight individuals, a higher alcohol consumption and a higher intake or processed meat than in Denmark^10,29^.

### Strength and limitations

Our report is currently the only updated evaluation of cancer burden attributable to lifestyle and environmental factors. The report provides a comprehensive risk profile, accommodates for changes in risk factor exposures and accounts for population trends in risk profiles and cancer incidence based on Danish registers. The approach was conservative since only Class I risk factors were considered, potential exposures may be missing and pending risk factor associations were not fully considered. This implies that our PAF values most likely underestimated the cancer burden^18,19^. Denmark has robust and population-based data on most of the studied risk factors, although recall bias and data source limitations should be recognized. Confidence intervals were not provided due to the multiple components in the PAF calculation. Applying the boundaries of the RR and risk factor exposure prevalence data to calculate the highest- and lowest-possible PAFs would be misleading, implying a precision that was not present as the PAFs were not adjusted for potential bias from the source data of the PAF calculations^10^.

Some risk factor–cancer type associations were considered not statistically significant in our updated evidence list of RRs, and the RR applied as 1 in the PAF calculations means that despite being a class I carcinogen it did not contribute to the PAF. In some cases, e.g. rare cancer types and low-prevalent risk factor exposures, the statistical precision was limited. Further, comparative evaluation across risk factors were limited by the RR and exposure prevalence data for the period of relevance. Adapted PAF data on occupational cancer from UK due to lack of national estimates may lead to over or underestimation depending on differences in occupational exposures between Denmark and UK. Similar the best available PAF data on cancer associated to HPV was also adapted, however cervix cancer, the largest associated cancer group, is recognized to be caused exclusively by HPV. Confounding is possible, e.g. related to tobacco smoking where former smokers’ risk was included, whereas the PAF estimates for alcohol consumption may be underestimated due to a control group including ex-drinkers^30^.

The ten-year latency period across all risk factors in our PAF calculation did not account for divergent times from risk exposure to development of cancer and the 10-year period chosen is relatively short, which also contributes to underestimates. Use of individual latency time for each cancer type in our calculations would not be systematic and would have reduced the possibility of comparison between risk factors. In addition, the selected latency matches with the length of follow-up time in the applied sources of RR and longer follow-up would yield a higher RR if the true latency was higher emphasizing that our PAF estimates are conservative. Tobacco smoking likely represent the largest underestimation of PAF due to the fixed latency period as smoking prevalence has continuously been falling during the past decades, and evidence that the latency period for tobacco-induced cancers is often longer than 10 years. When applying a 20-year latency period (using smoking rates from 1998^31^), the PAF for tobacco smoking increased by 1.5 pct. point compared with the results for 10-year latency.

Another source of underestimation was lack of consideration of synergistic effects. For instance, tobacco smoking and alcohol drinking have synergistic effects in relation to e.g. oesophageal cancer risk, and radon and smoking have synergistic effects on lung cancer risk. In both these examples, the effect of the combined exposure is considerable larger than the sum of the individual effects of the factors^32,33^. The RR applied in the calculations was typically adjusted, but control for confounding factors varies between risk factors. When summarizing PAFs of individual risk factors to calculate the combined total contribution, we avoid overestimation by applying PAFs sequentially only to the cases not attributed for by factors earlier in the sequence. PAFs for risk factor–cancer type combinations represent the fraction of a certain cancer type attributable to a certain risk factor in isolation, when the effect of other risk factors has been controlled for in the relative risk figure. Synergistic effects were not possible to include in our calculations, as the prevalence of combined risk factor exposure was generally not available. In addition, there is vice versa a possibility of double counting if synergistic effects were included as residual confounding in the RRs for individual risk factors, would likely lead to overestimating the PAF.

In general, the prevalence of exposure to risk factors obtained from surveys is prone to self-reporting bias leading to underestimation of exposure^34^.

### Projection

Future predictions of the development in PAFs can be estimated with the available knowledge of future trends in the development of risk factor exposure. During 2008–2018, the annual risk factor monitoring in Denmark showed that as in many countries overweight/obesity has increased, while tobacco smoking, alcohol consumption, and UV-radiation proxy measure (sunburn) has decreased slightly^35^. HPV infections are expected to decrease with the introduction of the vaccination program^36^ and a corresponding decrease in cervix cancer will follow. This development is likely to be reflected in future PAF calculations. Life expectancy and cancer survival are also increasing which may influence the development as well.

### Perspectives

The annual cost to society of the total burden of cancers diagnosed in 2013 in Denmark was estimated to 11.6 billion DKR (~ 1.5 billion €)^37^. A strengthened prevention effort is required to keep health costs under control. Increasing life expectancy needs to be accompanied by more healthy years.

The PAFs demonstrate a cross-sectional view of the avoidable number of cancer cases based on historic risk factor exposure prevalence, however, the reduction in risk factor prevalence required to avoid these cases may not be immediately achievable. Anderson et al. demonstrated for several risk factors how various introduced measures reduce the cancer incidence gradually over time^5–8,38–40^. Such modeling also includes e.g. the aging of populations and derived increases in cancer incidences and is a relevant supplement in the estimation of PAFs.

The class 2a classification of cancer risk factors, i.e. probably carcinogenic to humans, IARC)^19^ and strong evidence ‘probably increasing’ risk in humans, WCRF)^18^, include additional risk factors and already included risk factor associated with additional cancer types. These factors comprise a large group which will be essential to quantify as well and a group which in general is referred to as carcinogenic when communicating public health advice^18,19^. This group includes dietary fiber, red meat, alcohol consumption, environmental tobacco smoke, adult body fatness, physical activity, salt conservation, sugar-sweetened beverages, breastfeeding, and having been breastfed. The 70th World Health Assembly estimated that in 2030 the number of cancers diagnosed worldwide will have increased above 21 million with a large fraction being avoidable by risk reduction^41^.

## Conclusion

Based on exposure data primarily from 2008, 32.2% of the cancer burden in Denmark is estimated to be caused by class I carcinogens. Tobacco smoking is the single risk factor responsible for the largest proportion of avoidable cancer cases followed by UV exposure. Our estimates are conservative with consideration only of causal risk factors and a 10-year latency. Additional inclusion of class 2a carcinogens, consideration of synergistic effects and longer latency periods would further increase the fraction of cancer caused by modifiable risk factors. The knowledge presented provides an important basis for prioritization of future health policy.

## Supplementary Information

Supplementary Information.

## Data Availability

Data is available in the Supplementary Material.
